# A Rare Case of Clostridium difficile Enteritis: A Common Bug in an Uncommon Place

**DOI:** 10.7759/cureus.4519

**Published:** 2019-04-22

**Authors:** Amreet K Aujla, Leon D Averbukh, Alexander Potashinsky, Lisa Rossi

**Affiliations:** 1 Internal Medicine, University of Connecticut Health Center, Farmington, USA; 2 Gastroenterology, University of Connecticut Health Center, Farmington, USA; 3 Gastroenterology, Saint Francis Hospital, Hartford, USA

**Keywords:** clostridium difficile, c.difficile enteritis, ileostomy, crohns disease, chronic osteom

## Abstract

Clostridium difficile (CD), a bacterium responsible for causing 15%-25% of all cases of infectious diarrhea, is most commonly associated with infection of the colon. Rarely, though with increasing frequency, it has been noted to infect the small intestine in what is referred to as CD enteritis. We present the case of a patient who was diagnosed and treated for CD enteritis, review the pathophysiology behind the infection, and discuss the diagnostic and treatment options available to healthcare professionals.

## Introduction

Clostridium difficile infection (CDI) is a major identifiable cause of antibiotic-associated diarrhea and is responsible for 15%-25% of all cases of infectious diarrhea [1]. Since the turn of the twenty-first century, the incidence of CDI has increased significantly. Particularly affected are patients with inflammatory bowel disease (IBD) for whom CDI has been implicated as an IBD exacerbation factor in up to 5% of cases [1]. While generally manifesting as infectious colitis, CDI involving the small bowel, known as CD enteritis, is a rare condition associated with increased hospital length of stay and health care costs, poor patient quality of life, and a high mortality rate of approximately 30% [2-3]. We present the case of a patient who was diagnosed and treated for CD enteritis, review the pathophysiology behind the infection, and discuss the diagnostic and treatment options available to healthcare professionals.

## Case presentation

The patient is a 55-year-old Caucasian male, with a past medical history significant for Crohn’s disease status post total colectomy with end ileostomy several years prior, on mesalamine therapy, chronic osteomyelitis on suppressive therapy with doxycycline, and end-stage renal disease on hemodialysis, who initially presented to the emergency department with symptoms of increased ostomy output, crampy abdominal pain, nausea, dizziness, and generalized weakness. His symptoms began 48 hours prior to presentation while undergoing hemodialysis. Vital signs on arrival were notable for a temperature of 36.7°C, heart rate of 100 beats per minute, and blood pressure of 70/50 mmHg. On physical exam, the patient’s abdomen was diffusely tender to palpation without peritoneal signs. The ileostomy was viable with a small amount of fluid noted in the ostomy bag. Laboratory examination revealed a white blood cell (WBC) count of 10,900 cells/mm^3^, hemoglobin of 14 g/dL, platelet count of 695,000 platelets/mm^3^, and serum lactate of 2.2 mg/dL. Blood cultures were obtained in the emergency department, which showed no growth. Stool studies from the patient’s stoma output were significant for C. difficile. The patient was treated with intravenous normal saline and was started on oral metronidazole for CDI. The patient’s ostomy output subsequently improved and once he was hemodynamically stable, he was discharged with a prescription of oral metronidazole for a total of 10 days of antibiotic therapy.

**Figure 1:**
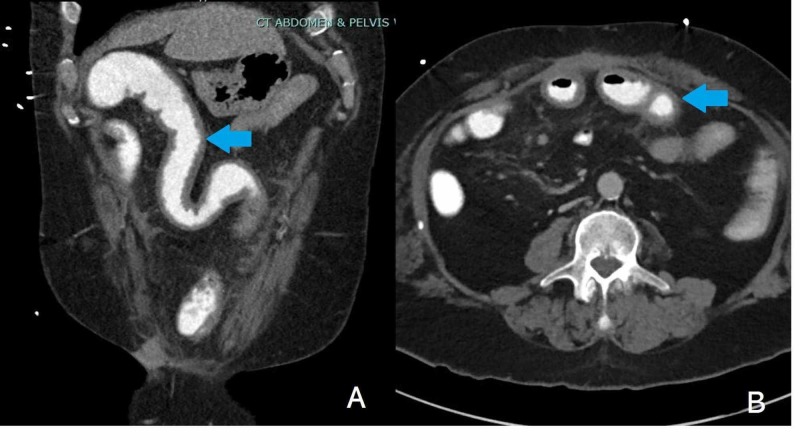
CT abdomen and pelvis with oral contrast showing bowel thickening with minimal stranding, suggestive of enteritis in coronal cut (blue arrow, A) and axial cut (blue arrow, B) CT: computed tomography

Three months later, the patient returned to the emergency department with a four-day history of increasing ostomy output with watery stools, epigastric abdominal pain, loss of appetite, and generalized weakness. One month prior to presentation, the patient had hip surgery and completed a 10-day course of doxycycline. Vital signs on arrival were notable for a temperature of 36.2°C, heart rate of 95 beats per minute, and blood pressure of 101/71 mmHg. On physical exam, his abdomen was tender to palpation in the epigastric region without peritoneal signs. The laboratory examination revealed a white blood cell count of 14,200 cells/mm^3^, hemoglobin of 10.2 g/dL, and a platelet count of 919,000 platelets/mm^3^. Inflammatory markers were also found to be elevated (C-reactive protein (CRP) 14.7 mg/L, estimated sedimentation rate (ESR) >130 mm/hr). A computed tomography (CT) abdomen and pelvis with oral contrast showed bowel thickening with minimal stranding, suggestive of enteritis along with enlarged peritoneal lymph nodes (Figure 1). Stool studies were positive for C. difficile and lactoferrin. The patient was started on oral vancomycin on which he subsequently improved. He was discharged to complete a 12-week course of antibiotic therapy.

## Discussion

C. difficile is a cytotoxin-producing, anaerobic, gram-positive bacterium, which was first isolated by Hall and O’Toole in 1935 from the stool of healthy neonates [4-5]. Colonization of the intestinal tract with C. difficile occurs through the fecal-oral route. Patients presenting with CDI generally experience symptoms of lower abdominal cramps as well as a greenish, foul-smelling, and watery (rather than bloody) diarrhea. C. difficile produces two distinct toxins: toxin A and toxin B. While initially believed to have distinctive actions, current literature supports both toxins producing cytotoxic and enteropathic effects. Toxins A and B disrupt the cytoskeleton of intestinal epithelial cells by the uridine diphosphate-glucose-dependent glycosylation of Ras and Rho proteins; these proteins are responsible for cell proliferation and cell morphology, respectively [6]. The diagnosis of CDI is established by the demonstration of C. difficile toxins in the stool and, less commonly, by C. difficile stool culture.

Isolated small-bowel involvement is exceedingly rare in cases of CDI but is more commonly seen in patients who've previously undergone colectomy [7]. Risk factors include increasing age, antibiotic use, proton pump inhibitor use, IBD, health care exposure, colectomy, and chronic comorbid conditions [3]. The pathogenesis of CD enteritis, however, remains somewhat unclear. Tsutaoka et al. suggested that after colectomy, the small-bowel bacterial flora may make the small intestine biome similar to that of the colon, thereby making it more susceptible to overgrowth with C. difficile [8]. According to Kralovich et al., the ileocecal valve inhibits the colonization of the small bowel with C. difficile via its peristaltic action, therefore, patients who undergo intestinal resections that include the ileocecal valve may be predisposed to developing CD enteritis [9]. The long duration between the primary intestinal operation and CD enteritis development may support the hypothesis that phenotypic changes may occur in the epithelium of the small intestine, altering the normal anatomy or fecal flow [7-9].

The diagnosis of CD enteritis requires a high index of suspicion. As many patients may not initially present with symptoms of CDI, a CT scan may provide supportive evidence. Ascites and a fluid-filled small bowel in the presence of mild mesenteric stranding, as demonstrated in our patient, is often consistent with CD enteritis [10]. Analogous to colonic infection, antibiotics such as oral or intravenous (IV) metronidazole, oral fidaxomicin, and oral vancomycin, along with supportive care, are the cornerstones of CD enteritis therapy.

Treatment is the same as for colonic C. difficile infection based on disease severity and is categorized as non-severe, severe, and fulminant CDI. Non-severe CDI is characterized by white blood cell count (WBC) <15,000 cells/mm^3^ and creatinine <1.5 mg/dL. Severe CDI is characterized by WBC >15,000 cells/mm^3^ and creatinine >1.5mg/dL. Fulminant CDI is characterized by hypotension, shock, ileus, or toxic megacolon. Treatment for non-severe CDI includes oral vancomycin or oral fidaxomicin for a 10-day course; however, it can be tailored according to the patient’s clinical status and comorbidities. Metronidazole is a second-line agent if oral vancomycin or oral fidaxomicin is not available. However, metronidazole should be avoided in patients who develop CDI in association with inflammatory bowel disease due to poor absorption [10]. For the first recurrence of CDI, treatment with oral vancomycin is appropriate. However, if there is a second recurrence, a vancomycin pulse-tapered regimen or fidaxomicin or vancomycin followed by a rifaximin regimen are recommended. For patients with a third recurrence, fecal microbiota transplantation is recommended. Severe CDI requires similar antibiotic management and early surgical intervention in order to reduce mortality [11].

## Conclusions

Though C. difficile involvement of the small bowel is rare, its incidence appears to be increasing. Risk factors for the development of CD enteritis include antibiotic use, proton pump inhibitor use, IBD, health care exposure, colectomy, and chronic comorbid conditions. A high index of suspicion and early identification of C. difficile in the small bowel is imperative for early intervention and includes stool cultures as well as CT imaging. The treatment of CD enteritis does not differ from that of CD colitis and includes antibiotics such as oral vancomycin, oral or IV metronidazole, and oral fidaxomicin, depending on the severity and recurrence status.
